# Hemisphere‐Level Comparison of Climate‐Driven Humpback Whale Breeding Migrations to the Eastern Pacific Off Costa Rica

**DOI:** 10.1002/ece3.73594

**Published:** 2026-05-04

**Authors:** Lili Pelayo‐González, Mario A. Pardo, Enrique Martínez‐Meyer, David Herra‐Miranda, Juan D. Pacheco‐Polanco, Sierra Goodman, Lenin Oviedo

**Affiliations:** ^1^ Laboratorio de Análisis Espaciales, Departamento de Zoología, Instituto de Biología, Universidad Nacional Autónoma de México, Ciudad Universitaria Mexico City Mexico; ^2^ Laboratorio de Macroecología Marina, Unidad Académica La Paz, Centro de Investigación Científica y de Educación Superior de Ensenada (CICESE)—Secretaría de Ciencia, Humanidades, Tecnología e Innovación (SECIHTI) La Paz Mexico; ^3^ Laboratorio de Ecología de Mamíferos Marinos Tropicales, Centro de Investigación de Cetáceos de Costa Rica (CEIC) Rincón de Osa Costa Rica

**Keywords:** Central America DPS, climate change, *Megaptera novaeangliae*, population connectivity, Southeastern Pacific DPS

## Abstract

This study evaluated humpback whale ( *Megaptera novaeangliae* ) occurrence off the Pacific coast of Costa Rica from 2001 to 2023, focusing on two Distinct Population Segments (DPSs), the Central America DPS and the Southeast Pacific DPS, which migrate from feeding grounds in opposite hemispheres. Using long‐term sightings and Bayesian time‐series and habitat models, we quantified the influence of absolute dynamic topography, sea surface temperature, and chlorophyll‐a on whale occurrence, and assessed whether environmental conditions increased the likelihood of convergence between the two DPSs. Extreme environmental conditions, including El Niño events and marine heatwaves, negatively affected whale counts, particularly for the Central America DPS, and a sustained decline occurred from 2007 to 2019 followed by recent recovery. Seasonal and interannual patterns indicated a potential temporal overlap of both DPSs between October and November, with anomalous warming in the Northern Hemisphere increasing the probability of overlap. These results demonstrate climate‐driven shifts in migratory patterns and connectivity, underscoring the need for continuous monitoring and improved tracking of both DPSs to inform conservation planning, whale‐watching management, and the protection of critical breeding habitats.

## Graphical abstract

This study analyzed humpback whale occurrence off the Pacific coast of Costa Rica from 2001 to 2023, focusing on the Central America and Southeast Pacific Distinct Population Segments, using long‐term sightings and Bayesian time‐series and habitat models. We found that environmental variability, including sea surface temperature, chlorophyll‐a, absolute dynamic topography, El Niño events, and marine heatwaves, strongly influenced whale occurrence, with marked negative effects and a prolonged decline from 2007 to 2019, followed by a partial recovery. Seasonal and interannual patterns suggest increasing temporal overlap between both population segments in October to November under anomalous warming, highlighting climate‐driven shifts in migration and the need for continued monitoring to inform conservation and management. Humpback whale illustration by Uko Gorter (usage rights granted to M.A. Pardo).

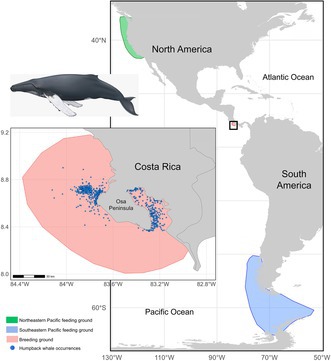

## 1 Introduction

Humpback whales ( *Megaptera novaeangliae* ) play an important role as large predators in marine ecosystems, undertaking seasonal migrations across vast distances between their feeding and breeding grounds (Rizzo and Schulte 2009; Meynecke et al. 2021). Currently, 14 distinct population segments (DPS) of humpback whales are recognized based on their breeding areas (NMFS and NOAA 2016). Nine of these are under a conservation status of “Least Concern”, one is categorized as “Threatened” (Mexico), and four are designated as “Endangered”, which include the DPSs of Central America, Cape Verde/Northwest Africa, Northwestern Pacific, and Arabian Sea (NMFS and NOAA 2016). The only locations registered worldwide where a possible convergence of DPSs from the northern and southern hemispheres occurs are the western coast of Africa (Cape Verde/Northwest Africa DPS and Gabon/Southwest Africa DPS) (Hazevoet et al. 2011) and the Pacific off Central America (Central America DPS and Southeastern Pacific DPS) (Acevedo and Smultea 1995).

In the coastal waters off Central America, humpback whales belonging to the subspecies *M. n. kuzira* (Jackson et al. 2014), arrive from the north during the early boreal winter, after spending the boreal summer feeding off the coasts of California, Oregon, and Washington, in the United States. Their wintering breeding destinations include Mexico and Central America (NMFS and NOAA 2021). Conversely, whales from the Southeastern Pacific DPS (*M. n. australis*) (Jackson et al. 2014) feed during the austral summer in the Southern Ocean, especially along the western Antarctic Peninsula and off southern Chile. Then, they migrate north to winter breeding grounds in coastal waters off South and Central America, from Ecuador to Costa Rica (Acevedo et al. 2013). Until recently, the coasts off Costa Rica were considered the boundary for both populations; however, there have been sightings of whales from the Central America DPS off Panama (Rasmussen et al. 2007), and reports of whales from the Southeastern Pacific DPS off Nicaragua (De Weerdt et al. 2020) and Mexico (González et al. 2023).

The Central America DPS is classified as “Endangered” under the United States Endangered Species Act due to its small population size (approximately 1400 individuals) (Curtis et al. 2022), unknown population trend, anthropogenic threats, and the occurrence of extreme climatic events affecting its feeding areas (NMFS and NOAA 2016). In contrast, the Southeastern Pacific DPS is considered at low risk, with an estimated population size of 6504 individuals and a positive growth trend (Johnston et al. 2020). While entanglement in fishing gear poses a threat to this population, there is no evidence that current rates of entanglement have a significant effect on the population's growth rate (NMFS and NOAA 2016).

Both populations feed and breed in environments characterized by complex oceanographic dynamics influenced by climate change, including interannual extreme weather events (Meynecke et al. 2021). The foraging habitat of the Central America DPS is influenced by the California Current System (CCS) (Fleming et al. 2016), whose productivity is modulated by climatic variability such as the Pacific Decadal Oscillation (PDO) (Di Lorenzo et al. 2008), which affects sea surface temperature and the North Pacific Gyre Oscillation (NPGO) (Di Lorenzo et al. 2009), associated with salinity and nutrient dynamics, as well as events like El Niño (Jacox et al. 2016) and marine heatwaves (Frölicher and Laufkötter 2018). These events have led to reductions in primary productivity, low prey availability for humpback whales (zooplankton and small pelagic fish) (Fleming et al. 2016), and harmful algal blooms (Ryan et al. 2017). In contrast, the foraging areas of the Southeastern Pacific DPS, located off southern Chile and the western Antarctic Peninsula (Dalla Rosa et al. 2008; Modest et al. 2021), are highly productive due to wind‐driven upwelling and seasonal sea ice melt. Off southern Chile, upwelling is strongest during austral spring and summer (Pinochet et al. 2019), and in Antarctica, the Antarctic Circumpolar Current promotes the upwelling of nutrient‐rich deep waters, which sustain Antarctic krill ( *Euphausia superba* ), the main prey of humpback whales in the region (Saba et al. 2014).

Over recent decades, regional atmospheric warming and changes in atmospheric circulation off the western Antarctic Peninsula have contributed to earlier seasonal sea‐ice retreat (Stammerjohn et al. 2008) and reduced primary productivity (Moreau et al. 2015; Kim et al. 2018), although local responses vary (Ferreira et al. 2024). The Southern Annular Mode (SAM) modulates climate in the Southern Hemisphere; negative SAM phases during winter and spring enhance phytoplankton growth and krill recruitment, whereas positive phases drive warm winds that cause early ice melt (Atkinson et al. 2019; Kawaguchi et al. 2024). Chlorophyll‐*a* anomalies occur every 4–6 years and are linked to negative SAM phases and El Niño events, corresponding to the lifespan of Antarctic krill (Saba et al. 2014). Although krill abundance has not declined over the past two decades, future increases in positive SAM phases may reduce krill biomass interannually, as lower summer chlorophyll‐*a* concentrations exert a greater effect than warming alone (Hill et al. 2013).

Changes in prey availability for humpback whales, whether due to declines in abundance or shifts in distribution toward offshore or more polar regions driven by climate change (Richardson 2008), affect their residency times and distribution patterns (Meynecke et al. 2021). Moreover, reduced prey availability (Mendes et al. 2023) may negatively impact their reproductive success and overall population dynamics (Seyboth et al. 2021). In recent years, the encounter rate of humpback whales from the Central America DPS off the coast of Costa Rica has declined, especially during marine warming events in the feeding areas off the United States (Pelayo‐González et al. 2022). However, it is still unclear how contrasting environmental conditions influence the occurrence of humpback whales from the Southeastern Pacific DPS off Central America. In recent years, mothers with calves from the Southeastern Pacific DPS have been recorded off Nicaragua (De Weerdt et al. 2020) and Mexico (González et al. 2023), which is outside the normal range of the population, and could reflect responses to changes in environmental conditions in their feeding and breeding areas.

If environmental conditions during the feeding season are unfavorable, humpback whales may not meet their energetic requirements and may consequently not migrate, delay or shorten migration, or even die due to starvation. Thus, the objective of this study was to analyze the occurrence of humpback whales in a critical breeding habitat off the coast of Costa Rica from 2001 to 2023 and its relationship with environmental conditions both locally and within the feeding areas of both populations in both hemispheres. We aimed to understand the multifaceted impacts of climate change on humpback whales inhabiting the eastern Pacific Ocean, as essential information for developing effective conservation strategies in both hemispheres. We expected that anomalous marine warming conditions would diminish the occurrence of humpback whales off Costa Rica, particularly for animals from the Central America DPS, due to a higher frequency of extreme climatic events in their feeding areas and their smaller population size compared to the Southeastern Pacific DPS.

## 2 Methods

### 2.1 Occurrences of Humpback Whales Off Costa Rica

**FIGURE 1:**
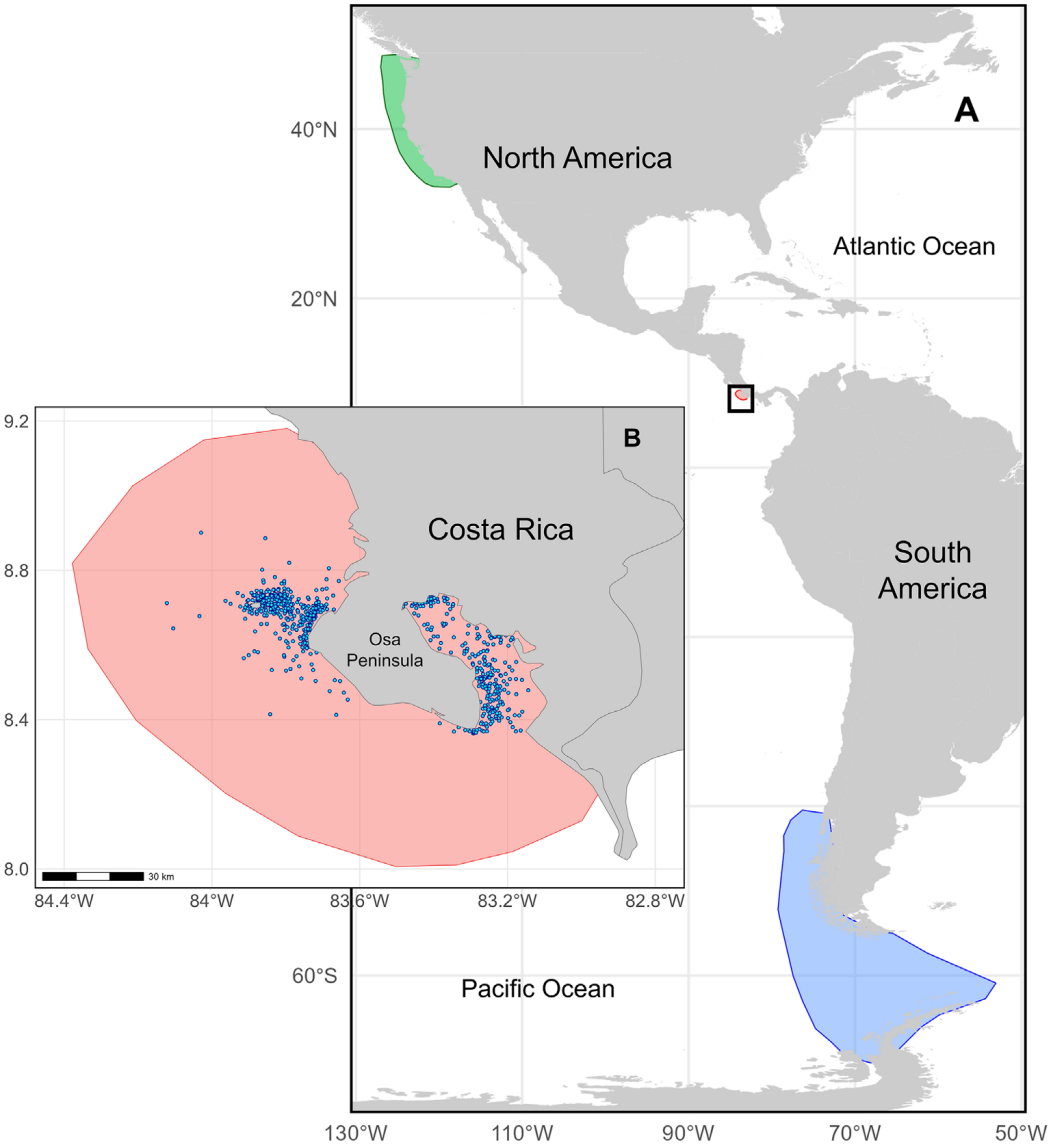
(A) Feeding areas of humpback whales from the Central American DPS (green) and the Southeastern Pacific DPS (blue). (B) Breeding habitat for both populations off Costa Rica (red) and their sightings from January 2001 to September 2023 (blue dots).

Humpback whale sightings were collected during cetacean observation surveys, conducted on opportunistic vessels with technicians and trained volunteers on board from January 2001 to September 2023 (Figure 1B; Figure S1). All whale data were collected under a research permit issued by the Ministry of Environment and Energy (Ministerio de Ambiente y Energía, MINAE) of Costa Rica, issued to L. Oviedo. Surveys were carried out using a 7‐m fiberglass boat equipped with a 115‐hp outboard engine. Although surveys targeted cetaceans, their frequency depended on logistical conditions and volunteer participation, resulting in an opportunistic sampling design rather than a systematic survey program. During these surveys, geographical position, detectability conditions, and the presence or absence of cetaceans were recorded every 30 min at established stations. The interpolated track between these positions was used as a proxy of survey effort. However, inconsistencies in the geographical records prevented us from using this proxy of survey effort in the final analysis. During humpback whale sightings, we recorded geographical position, group size, and group composition.

Because survey effort was not systematic and spatial coverage varied among surveys, the number of occurrences fluctuated among sampling days, likely reflecting differences in sampling effort rather than true count variability. To minimize such short‐term noise, we used the maximum number of occurrences detected in a single day within each 14‐day period as the response variable. This metric represents a conservative index of the minimum number of occurrences observed within each period. Out of 545 14‐day periods in the time series span, 234 (43%) had some sampling effort.

### 2.2 Environmental Analysis

Given their long‐term and global coverage availability, the absolute dynamic topography (ADT) of the ocean's surface, sea surface temperature (SST), and sea surface chlorophyll‐*a* concentration (CHL) were selected to address the influence of environmental changes on the occurrence of humpback whales in the critical breeding habitat off Costa Rica. The feeding polygons of interest were delimited following the general distribution patterns of the Southeastern Pacific DPS from published geographic locations of humpback whale sightings (Dalla Rosa et al. 2008; Modest et al. 2021), as well as from critical habitats previously defined for the Central America DPS (NMFS and NOAA 2021). The breeding polygon off Costa Rica was determined using the data from this study (Figure 1).

For the breeding region off Costa Rica, 14‐day period averages of ADT and SST were calculated from January 2001 to September 2023, as these variables indicate the heat content of the water column and surface, respectively, which has been proposed to affect neonate thermoregulation (Braithwaite et al. 2015) and therefore could be indices of breeding habitat quality. For the feeding areas, averages of ADT, SST, and CHL were calculated, as these variables could indicate oceanographic processes affecting food availability for humpback whales (Pardo et al. 2015; Fleming et al. 2016). Averages were estimated for the peak of the feeding seasons: April to August for the Central America DPS and October to February for the Southeastern Pacific DPS, accounting for a two‐month lag between environmental changes and subsequent impacts on phytoplankton and the availability of small pelagic fish (Takahashi and Checkley Jr. 2008). Finally, to identify long‐term trends and extreme events, we conducted a time series analysis for each environmental variable across each region.

#### 2.2.1 Absolute Dynamic Topography

Absolute dynamic topography (ADT) measures the ocean surface height relative to what it would be under gravitational influence alone. Because the ocean is affected by energy flows such as wind and ocean currents, its surface deviates from the geoid. ADT is calculated by adding the ocean level anomaly to the mean dynamic topography (Copernicus Marine Service Information 2023). High ADT values indicate an increase in water column volume, associated with heat‐content gain, which at the interannual scale would respond to warming events like El Niño; whereas low ADT values indicate a decrease in the water column volume, linked to cold events such as La Niña (Gourdeau et al. 2003; Pardo et al. 2015). Daily satellite images of ADT, level 3 with a spatial resolution of 0.25° × 0.25° were obtained from the E.U. Copernicus Marine Service (Copernicus Marine Service Information 2023). ADT averages were calculated for the feeding periods of each humpback whale population from April 2000 to February 2023.

#### 2.2.2 Sea Surface Temperature

We used daily satellite images of sea surface temperature (SST) with an original spatial resolution of 0.05° × 0.05° from the Pathfinder climate data record, acquired using the Advanced Very High‐Resolution Radiometer (AVHRR) aboard NOAA polar‐orbiting satellites from April 2000 to February 2023 (Saha et al. 2018). For this study, the resolution was reduced to 0.25° × 0.25° cells before extracting the desired polygons. Daily mean sea surface temperature (°C) was calculated for each area (Figure 1), and these values were subsequently aggregated to obtain historical averages for the feeding periods of each humpback whale population from April 2000 to February 2023.

#### 2.2.3 Sea Surface Chlorophyll‐*a*

Remotely sensed sea‐surface chlorophyll‐*a* concentration (CHL) data was obtained as eight‐day averages, level 3, with an original resolution of 0.0125° × 0.0125°. These were derived from in situ measurements and remote sensing reflectance using the Moderate Resolution Imaging Spectroradiometer (MODIS) onboard NASA's Aqua satellite (NASA Goddard Space Flight Center, Ocean Ecology Laboratory, Ocean Biology Processing Group 2014). For this study, the resolution was reduced to 0.25° × 0.25° cells before extracting the desired polygons. CHL averages were calculated for the feeding periods of each humpback whale population from July 2002 to April 2023, based on the availability of data for this variable.

### 2.3 Statistical Analysis

#### 2.3.1 Time‐Series of Whale Counts

A time series analysis was conducted on the humpback whale data to identify long‐term trend, seasonality, and interannual events. Several competing models were tested, including a potential long‐term trend (i.e., a simple linear effect) and a cyclic random‐walk random effect of the month of the year to assess seasonality. Additionally, autoregressive models of year (orders 1 to 6) were tested to isolate interannual variations as independent, identically distributed Gaussian random effects. Different complexity terms, including linear, quadratic, and cubic polynomials, as well as Poisson and negative binomial likelihood functions, were also evaluated (Maunder and Punt 2004).

The integrated nested Laplace approximation (INLA; Rue et al. 2009) was used to fit Bayesian hierarchical models, calculating approximations of the posterior marginal distributions of model parameters (Martino and Rue 2008; Gómez‐Rubio 2020). From all competing model alternatives, we chose the most robust based on the lowest value of the Watanabe‐Akaike information criterion (WAIC; Watanabe 2010). WAIC allows for a more precise capture of uncertainty, and is widely applied in mixed and hierarchical models, since it utilizes the entire posterior distribution of model parameters (Martino and Rue 2008; Gómez‐Rubio 2020). Goodness of fit was also evaluated independently by estimating a mean predictive integral transform (PIT), for which values very close to 0.5 indicate perfect fit. Additionally, the Bayesian R‐squared ($R_{B}^{2}$) was calculated, since it represents the proportion of posterior predictive variance explained by the model compared to the total variance of observed data (Gelman and van Dyk 2004; Martino and Rue 2008; Gómez‐Rubio 2020), and therefore, a measure of model accuracy. Best models were ranked according to their ɅWAIC values, and those within the same order of magnitude were averaged to account for model selection uncertainty. Model‐averaged predictions were obtained by combining posterior predictions from the selected models using WAIC‐based weights (Neil and Sitison 2024).

#### 2.3.2 Ecological Models

Mixed‐effects regression models were built using humpback whale counts as response variable of several combinations of environmental predictors, as well as others which also included the scales of temporal variations explained above. Feeding polygon averages were aligned with whale counts from the immediately following breeding season, while breeding polygon averages matched whale counts directly. In total, we tested 124 models containing uncorrelated variables and representing some potential ecological mechanisms (i.e., hypotheses). From the best model's predictions, we evaluated expected whale counts at the seasonal scale under three contrasting interannual scenarios around the breeding ground off Costa Rica: the anomalous warm conditions during 2015, the cold anomaly of 2001, and the neutral conditions that occurred during the rest of the years.

All data processing, analyses, and graphical representations were conducted in R (R Core Team 2022) using the following packages: *abind* (Plate and Heiberger 2016), *dplyr* (Wickham et al. 2023), *ggplot2* (Wickham et al. 2016), *ggsn* (Santos Baquero 2019), *INLA* (Rue et al. 2009), *lubridate* (Grolemund and Wickham 2011), *maptools* (Bivand and Lewin‐Koh 2023), *ncdf4* (Pierce 2017), *RColorBrewer* (Neuwirth 2022), *RNetCDF* (Michna and Woods 2023), *scales* (Wickham et al. 2023), *svMisc* (Grosjean 2022), and *viridis* (Garnier et al. 2023).

## 3 Results

### 3.1 Environmental Variables

#### 3.1.1 Absolute Dynamic Topography

The average ADT across the three regions showed an increasing trend (Figure S2). Mean ADT values were 48.9 ± 2.8 cm in the North Pacific feeding area, 69.9 ± 4.3 cm in the breeding area off Costa Rica, and −97.6 ± 3.3 cm in the South Pacific feeding area. The highest ADT anomalies in the North Pacific were recorded in 2014, followed by 2006 and 2023, while the lowest values occurred in 2013 and 2022. In the Costa Rica breeding area, the highest anomaly was observed in 2015 and the lowest in 2007. In the Southern Hemisphere, ADT exhibited positive anomalies between 2012 and 2015, and negative anomalies in 2001 and from 2021 to 2023 (Figure S3).

#### 3.1.2 Sea Surface Temperature

The average SST across the three regions showed an increasing trend (Figure S4). Mean SST values were 12.8°C ± 0.7°C in the North Pacific feeding area, 28.1°C ± 0.4°C in the breeding area off the coast of Costa Rica, and 8.72°C ± 0.5°C in the South Pacific feeding area. Similar temporal patterns were observed in the North Pacific and Costa Rica, with positive anomalies between 2003–2005 and 2014–2016, and lower values during 2008 and 2013. In contrast, in the South Pacific feeding area, the highest positive anomaly was recorded in 2023, while the lowest values occurred in 2015 and 2019 (Figure S5).

#### 3.1.3 Sea Surface Chlorophyll‐a Concentration

The average CHL showed contrasting patterns between regions, with a negative trend in the North Pacific feeding area and an increasing trend in the South Pacific feeding area (Figure S6). Mean CHL concentrations were 0.4 ± 1.2 mg m^−3^ in the North Pacific feeding area and 0.7 ± 1.1 mg m^−3^ in the South Pacific feeding area. In the North Pacific feeding area, the highest positive anomaly was observed in 2022, preceded by a period of positive anomalies between 2006 and 2011. The lowest negative anomaly occurred in 2004, with an extended period of negative anomalies from 2016 to 2021. In the Southern Hemisphere, positive anomalies were recorded in 2002, 2011, and 2020, while negative anomalies occurred during two main periods: 2003–2009 and 2012–2015 (Figure S7).

### 3.2 Time Series of Counts

**FIGURE 2:**
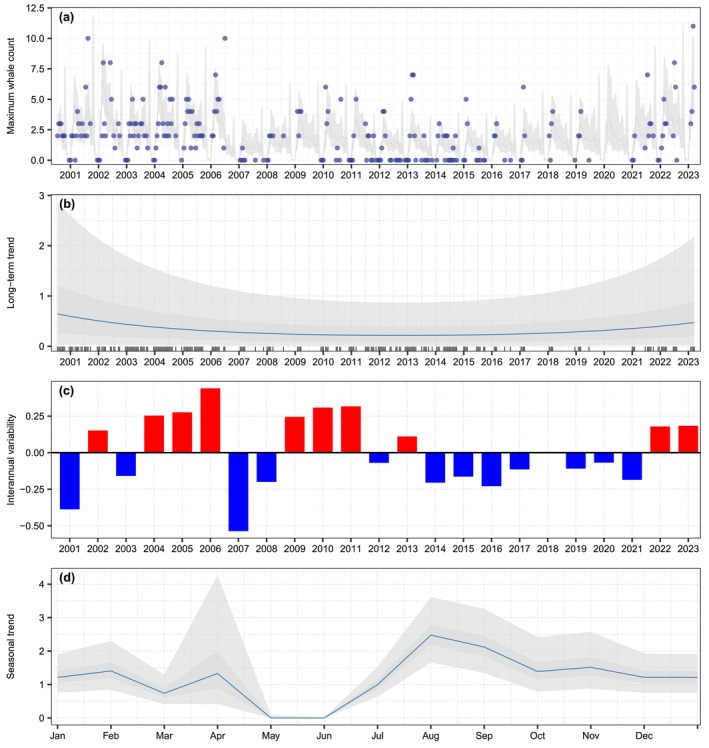
Time series of maximum daily humpback whale counts during a 14‐day period in their breeding habitat off Costa Rica and the partial effects of each scale of variation: (a) Observations (blue dots) and overall predictions from the model; (b) long‐term quadratic trend; (c) interannual variability (bars represent the median autoregressive random effect for each year); (d) seasonal cycle (values represent the posterior monthly random effect). The shaded areas in gray represent the 75%‐ and 95%‐credible intervals and the blue line indicates the median of the posterior distributions.

Time series analysis revealed a long‐term quadratic trend and a clear two‐peak seasonal pattern. Because the two first models showed similar support (Table 1), predictions were obtained using model averaging between them. The models differed only in the order of the autoregressive term indicating that yearly values were correlated with those up to 4–6 years in the past (Table 1; Figure 2b,c). The seasonal pattern showed two median peaks in counts during February–April and August–September. Remarkably, the median seasonal predictions showed complete absence of the species during May–June (Figure 2d).

**TABLE 1:** The ten most robust time‐series models of maximum daily humpback whale counts during a 14‐day period from 2001 to 2023 ($Y_{t}$), as a function of several scales of time.

No.	Model structure	PIT	$R_{B}^{2}$	WAIC	∆ WAIC
1	$Y_{t}=F_{t}+F_{t}^{2}+S_{m}+A_{6}Y_{t-6}$	0.515	0.373	778.235	0.000
2	$Y_{t}=F_{t}+F_{t}^{2}+S_{m}+A_{4}Y_{t-4}$	0.516	0.500	777.456	0.221
3	$Y_{t}=F_{t}+S_{m}+A_{5}Y_{t-5}$	0.514	0.431	779.443	1.208
4	$Y_{t}=F_{t}+S_{m}+A_{3}Y_{t-3}$	0.514	0.500	779.658	1.423
5	$Y_{t}=F_{t}+S_{m}+A_{6}Y_{t-6}$	0.514	0.451	779.722	1.487
6	$Y_{t}=F_{t}+F_{t}^{2}+S_{m}+A_{1}Y_{t-1}$	0.515	0.389	780.993	2.758
7	$Y_{t}=F_{t}+S_{m}+A_{2}Y_{t-2}$	0.507	0.500	782.224	3.989
8	$Y_{t}=F_{t}+S_{m}+A_{1}Y_{t-1}$	0.504	—	783.734	5.499
9	$Y_{t}=F_{t}+F_{t}^{2}+S_{m}+A_{2}Y_{t-2}$	0.498	0.500	785.566	7.331
10	$Y_{t}=F_{t}+F_{t}^{2}+S_{m}+A_{5}Y_{t-5}$	0.487	0.500	789.545	11.310

*Note:* The order of the models was based on the lowest Watanabe‐Akaike Information Criterion (∆WAIC). A mean predictive integral transform (PIT) close to 0.5 indicates better goodness of fit and high Bayesian R‐squared ($R_{B}^{2}$) values indicate high accuracy. Candidate models included as time scale terms: the 14‐day period count along the series ($F_{t}$), a cyclic random effect of the month of the year ($S_{m}$), and autoregressive random effects of year (orders 1 to 6) ($A_{i}Y_{t-i}$). All models shown utilized a negative binomial likelihood function.

### 3.3 Ecological Models

**FIGURE 3:**
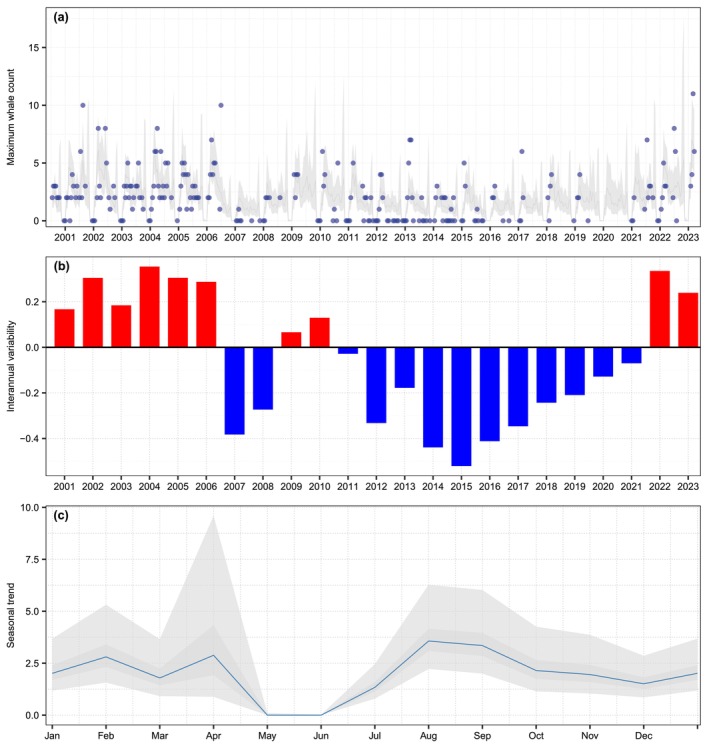
Time series of 14‐day period maximum counts of humpback whales in their breeding habitat off Costa Rica and the partial effects of covariates. (a) Ecological model; (b) Interannual variability; (c) Seasonal trend. Gray shaded areas represent 75% and 95% credibility intervals; the blue line indicates the median effect.

**FIGURE 4:**
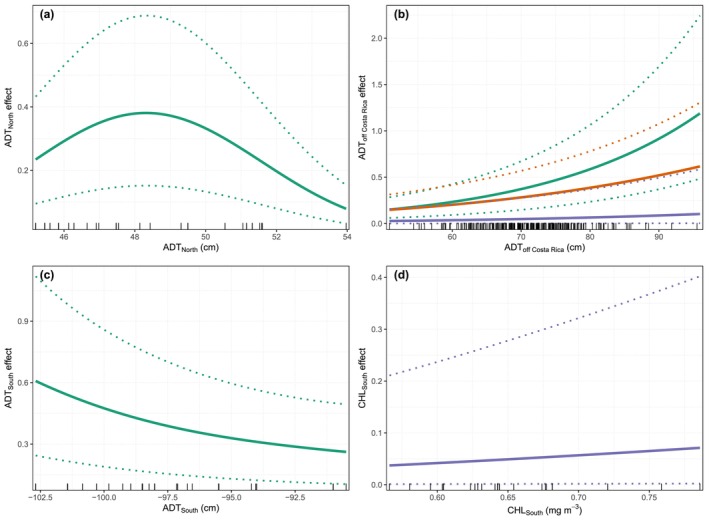
Partial effects of environmental covariates in the best ecological model. (a) the absolute dynamic topography (ADT) of the northern Pacific feeding area; (b) the ADT of the breeding area off Costa Rica; (c) the ADT of the southern Pacific feeding area; and (d) the CHL of the southern feeding area. The green lines represent the best model, the orange lines the second‐best model, and the purple lines the third‐best model.

The model‐averaged predictions based on the three best‐supported models incorporated ADT from the breeding area off Costa Rica, as well as from the northern and southern feeding areas, in addition to chlorophyll concentration (CHL) in the southern feeding area. All models included a seasonal component. The two top‐ranked models, which included ADT predictors, also incorporated a sixth‐order autoregressive structure for year, whereas the model including CHL retained a fourth‐order autoregressive structure (Table 2; Figure 3a). The latter indicated a decline in counts from 2011 to 2020 (Figure 3b). The seasonal cycle was very similar to that of the time‐series model (Figure 3c). Partial effects from the model‐averaged predictions indicated that ADT in the northern feeding area exhibited a quadratic relationship, with an optimum at intermediate values (approximately 47–49 cm). In the breeding area off Costa Rica, ADT showed a positive relationship with humpback whale occurrence. In the southern feeding area, ADT displayed a negative relationship, with lower ADT values associated with lower whale occurrence. In contrast, CHL in this region exhibited a positive relationship with whale occurrence (Figure 4).

**TABLE 2:** The ten best ecological models of humpback whale maximum daily counts in a 14‐day period ($Y_{t}$) from 2001 to 2023, as a function of environmental predictors in their critical feeding and breeding habitats.

No.	Model structure	PIT	$R_{B}^{2}$	WAIC	Ʌ WAIC
1	$Y_{t}={\mathrm{ADT}}_{\mathrm{CR}}+{\mathrm{ADT}}_{N}+{\mathrm{ADT}}_{N}^{2}+{\mathrm{ADT}}_{S}+{\mathrm{ADT}}_{S}^{2}+F_{t}+S_{m}+A_{6}Y_{t-6}$	0.513	0.438	773.717	0.000
2	$Y_{t}={\mathrm{ADT}}_{\mathrm{CR}}+S_{m}+A_{6}Y_{t-6}$	0.512	0.408	773.893	0.176
3	$Y_{t}={\mathrm{ADT}}_{\mathrm{CR}}+{\mathrm{CHL}}_{S}+S_{m}+A_{4}Y_{t-4}$	0.514	0.417	773.905	0.188
4	$Y_{t}={\mathrm{ADT}}_{\mathrm{CR}}+{\mathrm{ADT}}_{N}+{\mathrm{ADT}}_{S}+{\mathrm{CHL}}_{S}+S_{m}+A_{6}Y_{t-6}$	0.513	0.427	774.044	0.327
5	$Y_{t}={\mathrm{ADT}}_{\mathrm{CR}}+{\mathrm{ADT}}_{N}+{\mathrm{ADT}}_{S}+S_{m}+A_{4}Y_{t-4}$	0.513	0.419	774.060	0.343
6	$Y_{t}={\mathrm{ADT}}_{\mathrm{CR}}+{\mathrm{ADT}}_{\mathrm{CR}}^{2}+{\mathrm{ADT}}_{\mathrm{CR}}^{3}+{\mathrm{ADT}}_{N}+{\mathrm{ADT}}_{N}^{2}+{\mathrm{ADT}}_{N}^{3}+{\mathrm{ADT}}_{S}+{\mathrm{ADT}}_{S}^{2}+{\mathrm{ADT}}_{S}^{3}+S_{m}+A_{6}Y_{t-6}$	0.512	0.448	774.076	0.359
7	$Y_{t}={\mathrm{ADT}}_{\mathrm{CR}}+{\mathrm{ADT}}_{N}+{\mathrm{ADT}}_{S}+{\mathrm{CHL}}_{N}+{\mathrm{CHL}}_{S}+S_{m}+A_{6}Y_{t-6}$	0.515	0.429	774.081	0.364
8	$Y_{t}={\mathrm{ADT}}_{\mathrm{CR}}+{\mathrm{CHL}}_{S}+F_{t}+S_{m}+A_{3}Y_{t-3}$	0.514	0.417	774.361	0.644
9	$Y_{t}={\mathrm{ADT}}_{\mathrm{CR}}+S_{m}+A_{5}Y_{t-5}$	0.512	0.404	774.394	0.677
10	$Y_{t}={\mathrm{ADT}}_{\mathrm{CR}}+{\mathrm{ADT}}_{N}+S_{m}+A_{6}Y_{t-6}$	0.513	0.410	774.415	0.698

*Note:* The models are sorted according to the lowest Watanabe‐Akaike Information Criterion (∆WAIC), predictive integral transform (PIT) values close to 0.5 indicate good fit, and higher Bayesian *R*‐squared ($R_{B}^{2}$) values indicate better accuracy. The models included as predictors: the absolute dynamic topography in the breeding area off Costa Rica (${\mathrm{ADT}}_{\mathrm{CR}}$); the ADT in the feeding area of the North Pacific (${\mathrm{ADT}}_{N}$); the ADT in the feeding area of the South Pacific (${\mathrm{ADT}}_{S}$); the CHL in the South Pacific feeding area (${\mathrm{CHL}}_{S}$); a seasonal cyclic random effect of the month of the year ($S_{m}$); an autoregressive random effect of the year count with varying orders from 1 to 6 ($A_{i}Y_{t-i}$); and the number 14‐day periods in the time series ($F_{t}$). All models used a negative binomial likelihood function.

**FIGURE 5:**
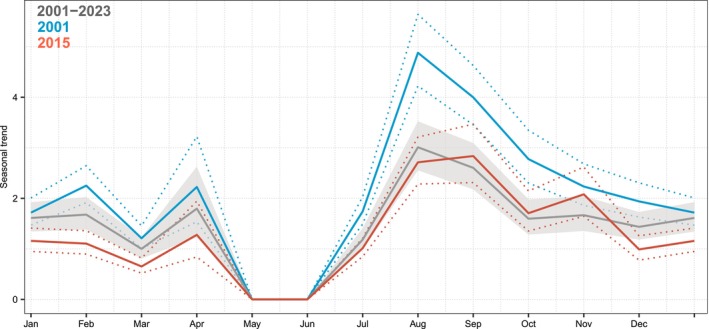
Seasonal trend of humpback whale counts off Costa Rica. Historical seasonality (gray line); seasonality under the lowest ADT values across the three areas (feeding and breeding areas) (blue line); and seasonality under the highest ADT values recorded in the northern feeding area and breeding area (red line). 95%‐credible intervals (shaded gray and dotted lines).

During the lowest ADT conditions of the time series (2001), humpback whale counts peaked in August, consistent with the peak observed in the long‐term seasonal cycle (2001–2023). In both cases, counts declined steadily after this peak. In contrast, during the highest ADT conditions (2015), the seasonal pattern showed a shift in peak timing, with maximum counts occurring in September, approximately 1 month later than the long‐term mean. Additionally, unlike 2001 and the long‐term average, whale counts in 2015 exhibited a secondary peak during October–November (Figure 5; Figure S3).

## 4 Discussion

This study examined the occurrence of humpback whales in breeding areas off Costa Rica from 2001 to 2023, revealing a long‐term quadratic trend. Counts also showed a sustained decline from 2011 to 2021 and fluctuations coinciding ENSO cycles and extreme marine heatwaves, notably the 2015–16 El Niño and the 2013–2016 North Pacific heatwave (Capotondi et al. 2022; Athanase et al. 2024). The lowest whale counts were recorded following El Niño events (e.g., 2002–2003, 2014–2016), while increases were observed during La Niña phases (e.g., 2005–2006, 2020–2023).

Population declines of humpback whales have been reported in the North Pacific, with a 20% drop from 2012 to 2021 (Cheeseman et al. 2024), and significant strandings (Santora et al. 2020) and reduced birth rates coinciding with warm marine anomalies during the same period (Frankel et al. 2022). In southeast Alaska, a 56% abundance decline occurred between 2013 and 2018 despite prior population growth (Gabriele et al. 2017, 2022). On the other hand, in the Western Antarctic Peninsula, the reproductive rate of humpback whales was lower in 2013, 2019, and 2020, and higher in 2014 and 2017, which was associated with krill availability (Pallin et al. 2023). In our study area, whale counts showed a quadratic pattern, with lower counts during the mid‐2010s and higher counts at the beginning and end of the time series. These periods of reduced counts broadly coincide with declines reported in North Pacific feeding areas, suggesting that inter‐annual changes may reflect population‐level variability affecting the Central America DPS.

Seasonal count predictions off Costa Rica indicated that humpback whales are present almost year‐round, except for May, which could serve as a dividing period between the two visiting populations. The last whales from the Central America DPS migrate north at the end of April, while whales from the Southeastern Pacific DPS begin to arrive in June and July (Figure 5). However, this division is not clear in November and December, when one would expect to see a lack of whales, allowing for the separation of those from the Southeastern Pacific DPS that have migrated to their feeding areas before whales from the Central America DPS arrive. Based on seasonal count predictions under cold conditions and across the entire study period, the decline observed between October and December may indicate the temporal separation between the two populations. The few individuals present in the area during this period could correspond either to Southeastern Pacific DPS whales that remained longer in the region or to the earliest Central America DPS arrivals off the coast of Costa Rica. In contrast, under anomalously warm conditions, seasonal predictions show a peak in early November. This pattern suggests that Southeastern Pacific DPS whales may remain in the area for an extended period, while Central America DPS whales arrive earlier, thereby increasing the potential for temporal overlap between the two populations.

In addition to potential changes in the whales' stay in their breeding area off Costa Rica, shifts in their distribution have also been observed. During marine heatwave events in the North Pacific, humpback whales from the Southern Hemisphere (Southeastern Pacific DPS) were observed in Nicaragua from July to October, peaking in August during 2015–2018, with a notable peak in 2017 (De Weerdt et al. 2020). This is considered unusual, as the geographic range of this population was previously thought to be limited to Costa Rica. Therefore, under warming conditions in the North Pacific, Southeastern Pacific DPS whales may now be observed in these areas, reflecting a response to environmental changes. These shifts in distribution and stay could influence the overlap of both populations across additional potential breeding areas, such as the northern coasts of Central America or Mexico.

Similarly, other marine mammal species from the Southern Hemisphere, such as the southern elephant seal ( *Mirounga leonina* ), have been observed outside their usual range. This species, typically limited to the Antarctic Ocean, has been seen along the coasts of Oaxaca, Chiapas, and Sonora, Mexico, between December and May during the period from 2020 to 2023, under environmental conditions associated with La Niña events (Romero‐Tenorio et al. 2023; Barba‐Acuña et al. 2024).

Despite the insights provided by this long‐term dataset, some limitations should be acknowledged, including the opportunistic nature of the surveys and the use of occurrence counts rather than direct estimates of abundance. Future research integrating standardized monitoring, photo‐identification, genetic analyses, and environmental data across the Central American region would help better understand population dynamics.

Therefore, we consider it necessary to implement and strengthen whale monitoring efforts along the coasts off Central America and southeastern Mexico, particularly during the summer, to track the occurrence of the Southeastern Pacific DPS, as these areas are typically studied in the context of the Central America DPS. Continuous monitoring of whale occurrence, together with environmental conditions, would allow for updating or determining current breeding and/or refuge areas for these DPSs, as well as guiding the planning of humpback whale tourism. Depending on environmental changes, the coasts off Central America and southeastern Mexico could potentially experience scenarios similar to those in Costa Rica (year‐round whale‐watching tourism). This would require re‐evaluating the potential effects of tourism activities on humpback whales and potentially updating permit conditions for whale‐watching operations to reduce impacts. Furthermore, it could provide an alternative to reduce anthropogenic pressure on the Central America DPS, which faces a higher threat level, and help protect critical breeding and nursery areas essential for its recovery.

## Author Contributions

**Lili Pelayo‐González:** conceptualization (equal), data curation (equal), formal analysis (equal), investigation (equal), methodology (equal), visualization (equal), writing – original draft (equal), writing – review and editing (equal). **Mario A. Pardo:** conceptualization (equal), formal analysis (equal), methodology (equal), supervision (equal), writing – review and editing (equal). **Enrique Martínez‐Meyer:** conceptualization (equal), supervision (equal), writing – review and editing (equal). **David Herra‐Miranda:** data curation (equal), writing – review and editing (equal). **Juan D. Pacheco‐Polanco:** data curation (equal), writing – review and editing (equal). **Sierra Goodman:** writing – review and editing (equal). **Lenin Oviedo:** conceptualization (equal), data curation (equal), funding acquisition (equal), investigation (equal), project administration (equal), resources (equal), supervision (equal), writing – review and editing (equal).

## Funding

Lili Pelayo‐González received a full Ph.D. scholarship from the Ministry of Science, Humanities, Technology and Innovation (SECIHTI; Grant No. 790305). This research was supported by the Centro de Investigación de Cetáceos de Costa Rica (CEIC), Vida Marina Foundation, and the Earthwatch Institute. M.A. Pardo received financial support from CICESE (Internal Project No. 691‐113).

## Disclosure

Our study brings together authors based in several countries, including researchers working within the region where the study was conducted. All authors were involved early in the development of the research questions and methodology to ensure that diverse perspectives and local knowledge informed the study design. Throughout the project, efforts were made to incorporate relevant literature produced by scientists from the region, including work published in the local language, and to maintain active communication with local stakeholders involved in marine mammal research and conservation.

## Conflicts of Interest

The authors declare no conflicts of interest.

## Supporting information


**FIGURE S1:** Average effort measured in kilometers conducted in the breeding area off Costa Rica. (A) Historical monthly average effort; (B) Annual average effort. Mean (blue points) and standard deviation (black bars).
**FIGURE S2:** Time series of historical averages of Absolute Dynamic Topography (ADT) across the three regions. Mean ADT values for humpback whale feeding and breeding areas are shown in black points. The northern Pacific feeding area is represented from April to August, the southern Pacific feeding area from October to February, and annual averages are shown for the breeding area off Costa Rica. Shaded gray areas represent the 75% and 95% credible intervals, and the median effect is indicated by the blue line.
**FIGURE S3:** Historical anomalies of Absolute Dynamic Topography (ADT) across the northern and southern feeding areas and the breeding area off Costa Rica.
**FIGURE S4:** Time series of historical averages of sea surface temperature (SST) across the three regions. Mean SST values for humpback whale feeding and breeding areas are shown in black points. The northern Pacific feeding area is represented from April to August, the southern Pacific feeding area from October to February, and annual averages are shown for the breeding area off Costa Rica. Shaded gray areas represent the 75% and 95% credible intervals, and the median effect is indicated by the blue line.
**FIGURE S5:** Historical anomalies of sea surface temperature (SST) across the northern and southern feeding areas and the breeding area off Costa Rica.
**FIGURE S5:** Historical anomalies of sea surface temperature (SST) across the northern and southern feeding areas and the breeding area off Costa Rica.
**FIGURE S6:** Time series of historical averages of sea surface chlorophyll‐a concentration (CHL) across the feeding areas. Mean CHL values for humpback whale feeding areas are shown (black points). The northern Pacific feeding area is represented from April to August, and the southern Pacific feeding area from October to February. Shaded gray areas represent the 75% and 95% credible intervals, and the median effect is indicated by the blue line.
**FIGURE S6:** Time series of historical averages of sea surface chlorophyll‐a concentration (CHL) across the feeding areas. Mean CHL values for humpback whale feeding areas are shown (black points). The northern Pacific feeding area is represented from April to August, and the southern Pacific feeding area from October to February. Shaded gray areas represent the 75% and 95% credible intervals, and the median effect is indicated by the blue line.
**FIGURE S7:** Historical anomalies of sea surface chlorophyll‐a concentration (CHL) across the northern and southern feeding areas.

## Data Availability

The data supporting the findings of this study are openly available in *Figshare* at https://doi.org/10.6084/m9.figshare.29936435.v1, reference number 57268976.

## References

[ece373594-bib-0001] Acevedo, A. , and M. A. Smultea . 1995. “First Records of Humpback Whales Including Calves at Golfo Dulce and Isla del Caño, Costa Rica, Suggesting Geographical Overlap of Northern and Southern Hemisphere Populations.” Marine Mammal Science 11, no. 4: 554–560. 10.1111/j.1748-7692.1995.tb00677.x.

[ece373594-bib-0002] Acevedo, J. , D. Haro , L. Dalla Rosa , et al. 2013. “Evidence of Spatial Structuring of Eastern South Pacific Humpback Whale Feeding Grounds.” Endangered Species Research 22, no. 1: 33–38. 10.3354/esr00536.

[ece373594-bib-0003] Athanase, M. , A. Sánchez‐Benítez , H. F. Goessling , F. Pithan , and T. Jung . 2024. “Projected Amplification of Summer Marine Heatwaves in a Warming Northeast Pacific Ocean.” Communications Earth & Environment 5, no. 1: 53. 10.1038/s43247-024-01212-1.

[ece373594-bib-0004] Atkinson, A. , S. L. Hill , E. A. Pakhomov , et al. 2019. “Krill ( *Euphausia superba* ) Distribution Contracts Southward During Rapid Regional Warming.” Nature Climate Change 9, no. 2: 142–147. 10.1038/s41558-018-0370-z.

[ece373594-bib-0005] Barba‐Acuña, I. D. , J. P. Gallo‐Reynoso , J. Á. Ortega‐Borchardt , et al. 2024. “New Sightings of Southern Elephant Seals ( *Mirounga leonina* ) in México: Citizen Science and Wildlife Dispersion.” Therya Notes 5, no. 2: 216–222.

[ece373594-bib-0071] Bivand, R. , and N. Lewin‐Koh . 2023. “maptools: Tools for Handling Spatial Objects (R package version 1.1‐8).” Retrieved from. https://CRAN.R‐project.org/package=maptools.

[ece373594-bib-0007] Braithwaite, J. E. , J. J. Meeuwig , and M. R. Hipsey . 2015. “Optimal Migration Energetics of Humpback Whales and the Implications of Disturbance. *Conservation* .” Physiology 3, no. 1: cov001. 10.12933/therya_notes-24-175. PMC4778463 27293686

[ece373594-bib-0008] Capotondi, A. , M. Newman , T. Xu , and E. Di Lorenzo . 2022. “An Optimal Precursor of Northeast Pacific Marine Heatwaves and Central Pacific El Niño Events.” Geophysical Research Letters 49, no. 5: e2021GL097350. 10.1029/2021GL097350.

[ece373594-bib-0009] Cheeseman, T. , J. Barlow , J. M. Acebes , et al. 2024. “Bellwethers of Change: Population Modelling of North Pacific Humpback Whales From 2002 Through 2021 Reveals Shift From Recovery to Climate Response.” Royal Society Open Science 11, no. 2: 231462. 10.1098/rsos.231462. 38420629 PMC10898971

[ece373594-bib-0010] Copernicus Marine Service Information . 2023. “Global Ocean Absolute Dynamic Topography (ADT) Product.” 10.48670/Moi-00149.

[ece373594-bib-0011] Curtis, K. A. , J. Calambokidis , K. Audley , M. G. Castaneda , and J. De Weerdt . 2022. “Abundance of Humpback Whales (Megaptera Novaeangliae) Wintering in Central America and Southern Mexico From a One‐Dimensional Spatial Capture‐Recapture Model (NOAA Technical Memorandum NMFS‐SWFSC‐661).” 10.25923/2k9h-zg41.

[ece373594-bib-0012] Dalla Rosa, L. , E. R. Secchi , Y. G. Maia , A. N. Zerbini , and M. P. Heide‐Jørgensen . 2008. “Movements of Satellite‐Monitored Humpback Whales on Their Feeding Ground Along the Antarctic Peninsula.” Polar Biology 31, no. 7: 771–781. 10.1007/s00300-007-0380-6.

[ece373594-bib-0013] De Weerdt, J. , E. A. Ramos , and T. Cheeseman . 2020. “Northernmost Records of Southern Hemisphere Humpback Whales ( *Megaptera novaeangliae* ) Migrating From the Antarctic Peninsula to the Pacific Coast of Nicaragua.” Marine Mammal Science 36, no. 3: 992–1000. 10.1111/mms.12684.

[ece373594-bib-0014] Di Lorenzo, E. , J. Fiechter , N. Schneider , et al. 2009. “Nutrient and Salinity Decadal Variations in the Central and Eastern North Pacific.” Geophysical Research Letters 36, no. 14: 38981. 10.1029/2009GL038981.

[ece373594-bib-0015] Di Lorenzo, E. , N. Schneider , K. M. Cobb , et al. 2008. “North Pacific Gyre Oscillation Links Ocean Climate and Ecosystem Change.” Geophysical Research Letters 35, no. 8: L08607. 10.1029/2007GL032838.

[ece373594-bib-0016] Ferreira, A. , C. R. Mendes , R. R. Costa , et al. 2024. “Climate Change Is Associated With Higher Phytoplankton Biomass and Longer Blooms in the West Antarctic Peninsula.” Nature Communications 15, no. 1: 6536. 10.1038/s41467-024-42388-0. PMC11297178 39095339

[ece373594-bib-0017] Fleming, A. H. , C. T. Clark , J. Calambokidis , and J. Barlow . 2016. “Humpback Whale Diets Respond to Variance in Ocean Climate and Ecosystem Conditions in the California Current.” Global Change Biology 22, no. 3: 1214–1224. 10.1111/gcb.13293. 26599719

[ece373594-bib-0018] Frankel, A. S. , C. M. Gabriele , S. Yin , and S. H. Rickards . 2022. “Humpback Whale Abundance in Hawai'i: Temporal Trends and Response to Climatic Drivers.” Marine Mammal Science 38, no. 1: 118–138. 10.1111/mms.12846.

[ece373594-bib-0019] Frölicher, T. L. , and C. Laufkötter . 2018. “Emerging Risks From Marine Heat Waves.” Nature Communications 9: 650. 10.1038/s41467-018-03163-6. PMC5811532 29440658

[ece373594-bib-0020] Gabriele, C. M. , C. L. Amundson , J. L. Neilson , J. M. Straley , C. S. Baker , and S. L. Danielson . 2022. “Sharp Decline in Humpback Whale ( *Megaptera novaeangliae* ) Survival and Reproductive Success in Southeastern Alaska During and After the 2014–2016 Northeast Pacific Marine Heatwave.” Mammalian Biology 102, no. 4: 1113–1131. 10.1007/s42991-022-00240-4.

[ece373594-bib-0021] Gabriele, C. M. , J. L. Neilson , J. M. Straley , C. S. Baker , J. A. Cedarleaf , and J. F. Saracco . 2017. “Natural History, Population Dynamics, and Habitat Use of Humpback Whales Over 30 Years on an Alaska Feeding Ground.” Ecosphere 8, no. 1: e01641. 10.1002/ecs2.1641.

[ece373594-bib-0022] Garnier, S. , N. Ross , R. Rudis , A. P. Camargo , M. Sciaini , and C. Scherer . 2023. “Viridis(Lite)—Colorblind‐Friendly Color Maps for R (Viridis Package Version 0.6.4).”

[ece373594-bib-0023] Gelman, A. , and D. A. van Dyk . 2004. “Bayesian Measures of Model Complexity and Fit.” Journal of the Royal Statistical Society: Series B 64, no. 4: 583–639. 10.1111/j.1467-9868.2004.02022.x.

[ece373594-bib-0024] Gómez‐Rubio, V. 2020. Bayesian Inference With INLA. Chapman and Hall/CRC. 10.1201/9780429323875.

[ece373594-bib-0025] González, M. L. , C. D. Ortega‐Ortiz , F. R. Elorriaga‐Verplancken , et al. 2023. “A Mother–Calf Humpback Whale ( *Megaptera novaeangliae* ) Pair From the Southeast Pacific Population Sighted in Mexican Waters.” Aquatic Mammals 49, no. 2: 208–216. 10.1578/AM.49.2.2023.208.

[ece373594-bib-0026] Gourdeau, L. , J. M. Lemoine , M. H. Rio , and F. Hernandez . 2003. “Estimating Mean Dynamic Topography in the Tropical Pacific Ocean From Gravity and Altimetry Satellites.” Geophysical Research Letters 30, no. 20: 18119. 10.1029/2003GL018119.

[ece373594-bib-0027] Grolemund, G. , and H. Wickham . 2011. “Dates and Times Made Easy With Lubridate.” Journal of Statistical Software 40: 1–25. 10.18637/jss.v040.i03.

[ece373594-bib-0028] Grosjean, P. 2022. SciViews‐R. UMONS. https://www.sciviews.org/SciViews‐R/.

[ece373594-bib-0029] Hazevoet, C. J. , B. Gravanita , P. López Suárez , and F. W. Wenzel . 2011. “Seasonality of Humpback Whale *Megaptera novaeangliae* (Borowski, 1781) Records in Cape Verde Seas: Evidence for the Occurrence of Stocks From Both Hemispheres.” Zoologia Caboverdiana 2, no. 1: 25–29.

[ece373594-bib-0030] Hill, S. L. , T. Phillips , and A. Atkinson . 2013. “Potential Climate Change Effects on the Habitat of Antarctic Krill in the Weddell Quadrant of the Southern Ocean.” PLoS One 8, no. 8: e72246. 10.1371/journal.pone.0072246. 23991072 PMC3749108

[ece373594-bib-0031] Jackson, J. A. , D. J. Steel , P. Beerli , et al. 2014. “Global Diversity and Oceanic Divergence of Humpback Whales ( *Megaptera novaeangliae* ).” Proceedings of the Royal Society B: Biological Sciences 281, no. 1786: 20133222. 10.1098/rspb.2013.3222. PMC4046397 24850919

[ece373594-bib-0032] Jacox, M. G. , E. L. Hazen , K. D. Zaba , et al. 2016. “Impacts of the 2015–2016 El Niño on the California Current System: Early Assessment and Comparison to Past Events.” Geophysical Research Letters 43, no. 13: 7072–7080. 10.1002/2016GL069716.

[ece373594-bib-0033] Johnston, S. J. , A. N. Zerbini , and D. S. Butterworth . 2020. “A Bayesian Approach to Assess the Status of Southern Hemisphere Humpback Whales ( *Megaptera novaeangliae* ) With an Application to Breeding Stock G.” Journal of Cetacean Research and Management 21: 309–317. 10.47536/jcrm.v21i1.234.

[ece373594-bib-0034] Kawaguchi, S. , A. Atkinson , D. Bahlburg , et al. 2024. “Climate Change Impacts on Antarctic Krill Behaviour and Population Dynamics.” Nature Reviews Earth & Environment 5, no. 1: 43–58. 10.1038/s43017-023-00504-y.

[ece373594-bib-0035] Kim, H. , H. W. Ducklow , D. Abele , et al. 2018. “Inter‐Decadal Variability of Phytoplankton Biomass Along the Coastal West Antarctic Peninsula.” Philosophical Transactions of the Royal Society A 376, no. 2122: 20170174. 10.1098/rsta.2017.0174. PMC5954473 29760117

[ece373594-bib-0036] Martino, S. , and H. Rue . 2008. “Implementing Approximate Bayesian Inference Using Integrated Nested Laplace Approximation: A Manual for the INLA Program.” https://www.r‐inla.org.

[ece373594-bib-0037] Maunder, M. N. , and A. E. Punt . 2004. “Standardizing Catch and Effort Data: A Review of Recent Approaches.” Fisheries Research 70, no. 2–3: 141–159. 10.1016/j.fishres.2004.08.002.

[ece373594-bib-0038] Mendes, C. R. B. , R. R. Costa , A. Ferreira , et al. 2023. “Cryptophytes: An Emerging Algal Group in the Rapidly Changing Antarctic Peninsula Marine Environments.” Global Change Biology 29, no. 7: 1791–1808. 10.1111/gcb.16575. 36656050

[ece373594-bib-0039] Meynecke, J. O. , J. de Bie , J. L. M. Barraqueta , et al. 2021. “The Role of Environmental Drivers in Humpback Whale Distribution, Movement and Behavior: A Review.” Frontiers in Marine Science 8: 720774. 10.3389/fmars.2021.720774.

[ece373594-bib-0072] Michna, P. , and M. Woods . 2023. “RNetCDF: Interface to ‘NetCDF’ datasets (R package version 2.6‐2).” Retrieved from. https://CRAN.R‐project.org/package=RNetCDF.

[ece373594-bib-0040] Modest, M. , L. Irvine , V. Andrews‐Goff , et al. 2021. “First Description of Migratory Behavior of Humpback Whales From an Antarctic Feeding Ground to a Tropical Calving Ground.” Animal Biotelemetry 9, no. 1: 1–16. 10.1186/s40317-021-00248-w.

[ece373594-bib-0041] Moreau, S. , B. Mostajir , S. Bélanger , et al. 2015. “Climate Change Enhances Primary Production in the Western Antarctic Peninsula.” Global Change Biology 21, no. 6: 2191–2205. 10.1111/gcb.12870. 25626857

[ece373594-bib-0042] NASA Goddard Space Flight Center, Ocean Ecology Laboratory, Ocean Biology Processing Group . 2014. “MODIS‐Aqua Ocean Color Data; NASA Goddard Space Flight Center, Ocean Ecology Laboratory, Ocean Biology Processing Group.” 10.5067/AQUA/MODIS_OC.2014.

[ece373594-bib-0043] National Marine Fisheries Service (NMFS), National Oceanic and Atmospheric Administration (NOAA), & Commerce . 2016. Endangered and Threatened Species; Identification of 14 Distinct Population Segments of the Humpback Whale ( *Megaptera novaeangliae* ) and Revision of Species‐Wide Listing. Vol. 81, 62259. Department of Commerce, NOAA.

[ece373594-bib-0044] National Marine Fisheries Service (NMFS), National Oceanic and Atmospheric Administration (NOAA), & Commerce . 2021. Endangered and Threatened Wildlife and Plants: Designating Critical Habitat for the Central America, Mexico, and Western North Pacific Distinct Population Segments of Humpback Whales. Vol. 86. Department of Commerce, NOAA. https://www.fisheries.noaa.gov/species/humpback‐whale#conservationmanagement.

[ece373594-bib-0045] Neil, E. T. , and J. W. Sitison . 2024. “Improved Information Criteria for Bayesian Model Averaging in Lattice Field Theory.” Physical Review D 109, no. 1: 14510. 10.1103/PhysRevD.109.014510.

[ece373594-bib-0046] Neuwirth, E. 2022. “RColorBrewer: ColorBrewer Palettes (R Package Version 1.1‐3).”

[ece373594-bib-0047] Pallin, L. J. , N. M. Kellar , D. Steel , et al. 2023. “A Surplus No More? Variation in Krill Availability Impacts Reproductive Rates of Antarctic Baleen Whales.” Global Change Biology 29: 2108–2121. 10.1111/gcb.16754. 36644792

[ece373594-bib-0048] Pardo, M. A. , T. Gerrodette , E. Beier , et al. 2015. “Inferring Cetacean Population Densities From the Absolute Dynamic Topography of the Ocean in a Hierarchical Bayesian Framework.” PLoS One 10, no. 3: e0120727. 10.1371/journal.pone.0120727. 25785692 PMC4364891

[ece373594-bib-0049] Pelayo‐González, L. , D. Herra‐Miranda , J. D. Pacheco‐Polanco , H. M. Guzmán , S. Goodman , and L. Oviedo . 2022. “Decreases in Encounter Rate of Endangered Northeast Pacific Humpback Whales in Southern Costa Rica: Possible Changes in Migration Pattern due to Warming Events.” Frontiers in Marine Science 9: 927276. 10.3389/fmars.2022.927276.

[ece373594-bib-0050] Pierce, D. 2017. “Interface to Unidata netCDF (Version 4 or Earlier) Format Data Files (R Package Version 1).”

[ece373594-bib-0051] Pinochet, A. , J. Garcés‐Vargas , C. Lara , and F. Olguín . 2019. “Seasonal Variability of Upwelling Off Central‐Southern Chile.” Remote Sensing 11, no. 15: 1737. 10.3390/rs11151737.

[ece373594-bib-0070] Plate, T. , and R. Heiberger . 2016. “abind: Combine Multidimensional Arrays (R package version 1.4‐5).” Retrieved from. https://CRAN.R‐project.org/package=abind.

[ece373594-bib-0052] R Core Team . 2022. R: A Language and Environment for Statistical Computing. R Foundation for Statistical Computing. https://www.R‐project.org/.

[ece373594-bib-0053] Rasmussen, K. , D. M. Palacios , J. Calambokidis , et al. 2007. “Southern Hemisphere Humpback Whales Wintering Off Central America: Insights From Water Temperature Into the Longest Mammalian Migration.” Biology Letters 3, no. 3: 302–305. 10.1098/rsbl.2007.0099. 17412669 PMC2390682

[ece373594-bib-0054] Richardson, A. J. 2008. “In Hot Water: Zooplankton and Climate Change.” ICES Journal of Marine Science 65, no. 3: 279–295. 10.1093/icesjms/fsn028.

[ece373594-bib-0055] Rizzo, L. Y. , and D. Schulte . 2009. “A Review of Humpback Whales' Migration Patterns Worldwide and Their Consequences to Gene Flow.” Journal of the Marine Biological Association of the United Kingdom 89, no. 5: 995–1002. 10.1017/S0025315409000400.

[ece373594-bib-0056] Romero‐Tenorio, A. , F. R. E. Verplancken , J. P. Gallo‐Reynoso , L. A. Álvarez‐Márquez , and I. D. Barba‐Acuña . 2023. “Records of Southern Elephant Seals ( *Mirounga leonina* ) in the Southern Mexican Pacific.” Latin American Journal of Aquatic Mammals 18, no. 2: 207–211. 10.5597/lajam00227.

[ece373594-bib-0057] Rue, H. , S. Martino , and N. Chopin . 2009. “Approximate Bayesian Inference for Latent Gaussian Models by Using Integrated Nested Laplace Approximations.” Journal of the Royal Statistical Society, Series B: Statistical Methodology 71, no. 2: 319–392. 10.1111/j.1467-9868.2008.00700.x.

[ece373594-bib-0058] Ryan, J. P. , R. M. Kudela , J. M. Birch , et al. 2017. “Causality of an Extreme Harmful Algal Bloom in Monterey Bay, California, During the 2014–2016 Northeast Pacific Warm Anomaly.” Geophysical Research Letters 44, no. 11: 5571–5579. 10.1002/2017GL073324.

[ece373594-bib-0059] Saba, G. K. , W. R. Fraser , V. S. Saba , et al. 2014. “Winter and Spring Controls on the Summer Food Web of the Coastal West Antarctic Peninsula.” Nature Communications 5, no. 1: 4318. 10.1038/ncomms5318. 25000452

[ece373594-bib-0060] Saha, K. , X. Zhao , H. Zhang , et al. 2018. AVHRR Pathfinder Version 5.3 Level 3 Collated (L3C) Global 4 Km Sea Surface Temperature for 1981‐Present [Dataset]. NOAA National Centers for Environmental Information. 10.7289/v52j68xx.

[ece373594-bib-0061] Santora, J. A. , N. J. Mantua , I. D. Schroeder , et al. 2020. “Impacts of El Niño Events on the California Current Pelagic Ecosystem: A Synthesis of Insights From Long‐Term Ecological Research.” Frontiers in Marine Science 7: 42. 10.3389/fmars.2020.00042.

[ece373594-bib-0062] Santos Baquero, O. 2019. “Ggsn: North Symbols and Scale Bars for Maps Created With ‘ggplot2’ or ‘ggmap’ (R Package Version 0.5.0).” https://CRAN.R‐project.org/package=ggsn.

[ece373594-bib-0063] Seyboth, E. , F. Félix , M.‐A. Lea , et al. 2021. “Influence of Krill ( *Euphausia superba* ) Availability on Humpback Whale ( *Megaptera novaeangliae* ) Reproductive Rate.” Marine Mammal Science 37, no. 4: 1498–1506. 10.1111/mms.12805.

[ece373594-bib-0064] Stammerjohn, S. E. , D. G. Martinson , R. C. Smith , X. Yuan , and D. Rind . 2008. “Trends in Antarctic Annual Sea Ice Retreat and Advance and Their Relation to El Niño–Southern Oscillation and Southern Annular Mode Variability.” Journal of Geophysical Research: Oceans 113, no. C3: 4269. 10.1029/2007JC004269.

[ece373594-bib-0065] Takahashi, M. , and D. M. Checkley Jr. 2008. “Growth and Survival of Pacific Sardine ( *Sardinops sagax* ) in the California Current Region.” Journal of Northwest Atlantic Fishery Science 41: 129–136. 10.2960/J.v41.m632.

[ece373594-bib-0066] Watanabe, S. 2010. “Asymptotic Equivalence of Bayes Cross Validation and Widely Applicable Information Criterion in Singular Learning Theory.” Journal of Machine Learning Research 11: 3571–3594.

[ece373594-bib-0068] Wickham, H. , W. Chang , and M. H. Wickham . 2016. “Package ‘ggplot2’. Create Elegant Data Visualisations Using the Grammar of Graphics (Version 2.1, 1–189).”

[ece373594-bib-0069] Wickham, H. , T. Pedersen , and D. Seidel . 2023. “Scales: Scale Functions for Visualization (R Package Version 1.3.0).” https://CRAN.R‐project.org/package=scales.

