# Supplementary material for: Hemisphere‐Level Comparison of Climate‐Driven Humpback Whale Breeding Migrations to the Eastern Pacific Off Costa Rica
Source: Ecol Evol. 2026 May 4;16(5):e73594. doi: 10.1002/ece3.73594 (PMC13139631; doi:10.1002/ece3.73594)
Supplement: Supplementary file 1 — FIGURE S1: Average effort measured in kilometers conducted in the breeding area off Costa Rica. (A) Historical monthly average effort; (B) Annual average effort. Mean (blue points) and standard deviation (black bars). FIGURE S2: Time series of historical averages of Absolute Dynamic Topography (ADT) across the three regions. Mean ADT values for humpback whale feeding and breeding areas are shown in black points. The northern Pacific feeding area is represented from April to August, the southern Pacific feeding area from October to February, and annual averages are shown for the breeding area off Costa Rica. Shaded gray areas represent the 75% and 95% credible intervals, and the median effect is indicated by the blue line. FIGURE S3: Historical anomalies of Absolute Dynamic Topography (ADT) across the northern and southern feeding areas and the breeding area off Costa Rica. FIGURE S4: Time series of historical averages of sea surface temperature (SST) across the three regions. Mean SST values for humpback whale feeding and breeding areas are shown in black points. The northern Pacific feeding area is represented from April to August, the southern Pacific feeding area from October to February, and annual averages are shown for the breeding area off Costa Rica. Shaded gray areas represent the 75% and 95% credible intervals, and the median effect is indicated by the blue line. FIGURE S5: Historical anomalies of sea surface temperature (SST) across the northern and southern feeding areas and the breeding area off Costa Rica. FIGURE S5: Historical anomalies of sea surface temperature (SST) across the northern and southern feeding areas and the breeding area off Costa Rica. FIGURE S6: Time series of historical averages of sea surface chlorophyll‐a concentration (CHL) across the feeding areas. Mean CHL values for humpback whale feeding areas are shown (black points). The northern Pacific feeding area is represented from April to August, and the southern Pacific feeding area [file ECE3-16-e73594-s001.pdf]

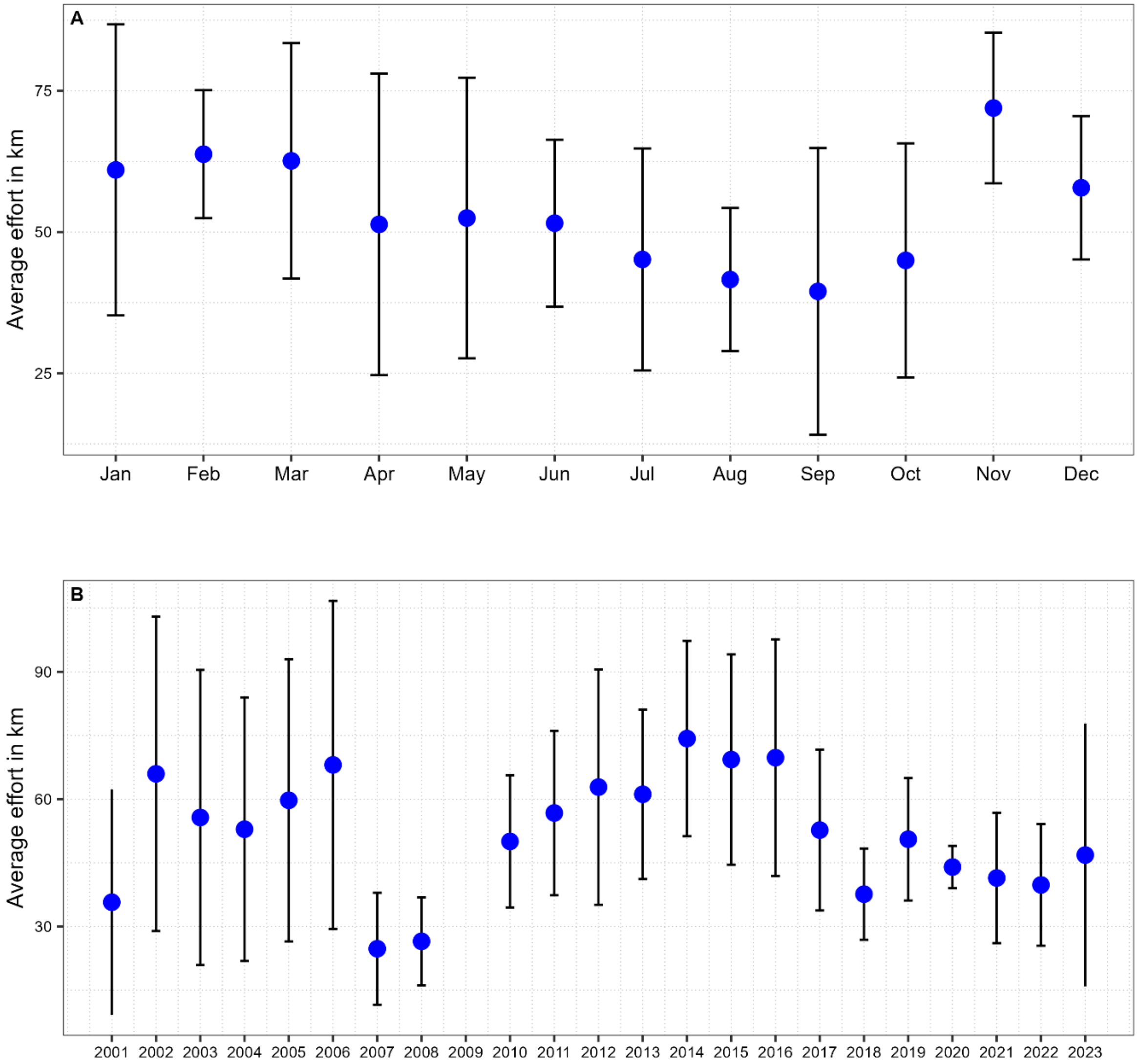

FIGURE S1. Average effort measured in kilometers conducted in the breeding area off Costa Rica. (A) Historical monthly average effort; (B) Annual average effort. Mean (blue points) and standard deviation (black bars).

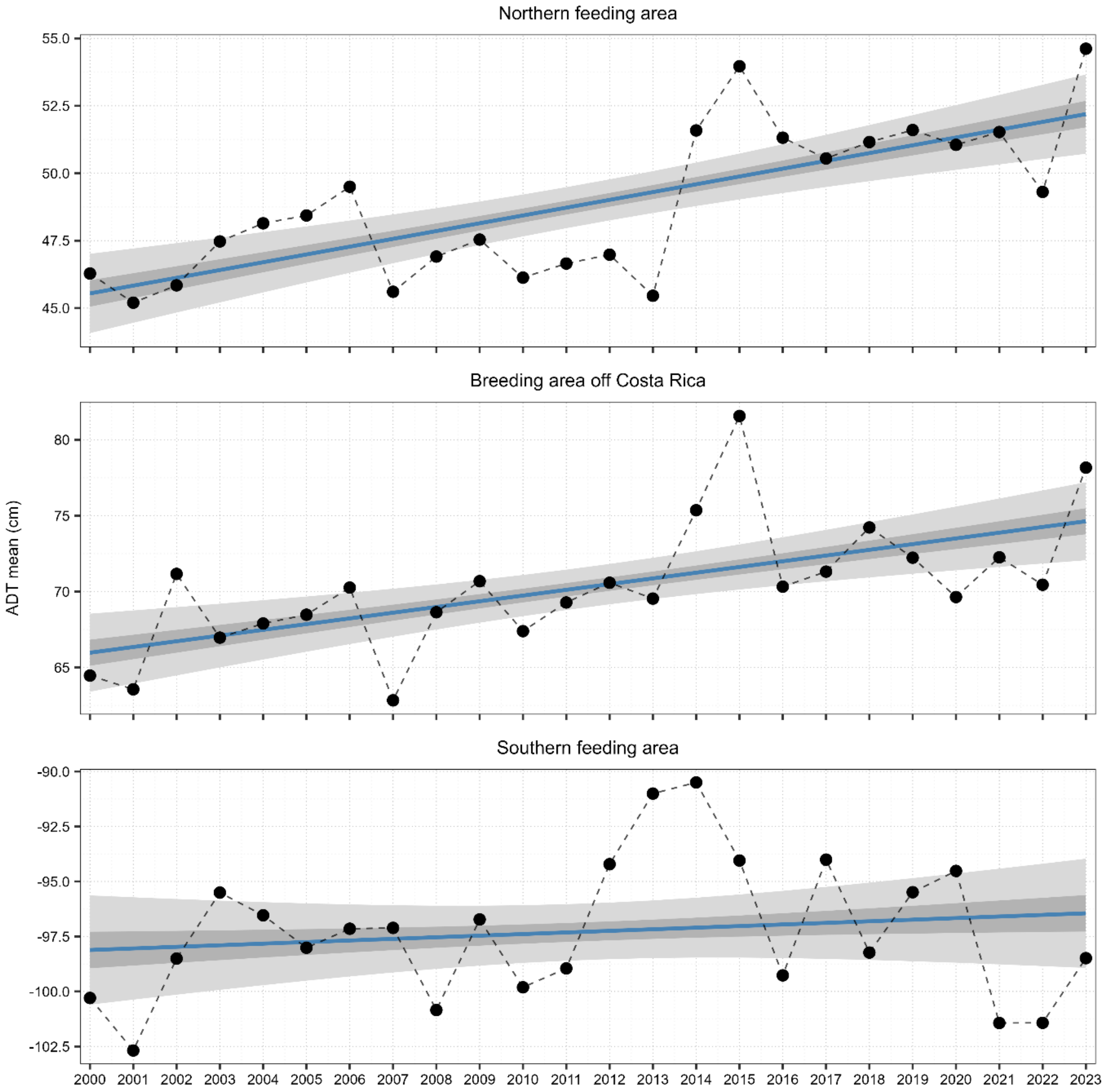

FIGURE S2. Time series of historical averages of Absolute Dynamic Topography (ADT) across the three regions. Mean ADT values for humpback whale feeding and breeding areas are shown in black points. The northern Pacific feeding area is represented from April to August, the southern Pacific feeding area from October to February, and annual averages are shown for the breeding area off Costa Rica. Shaded gray areas represent the 75% and 95% credible intervals, and the median effect is indicated by the blue line.

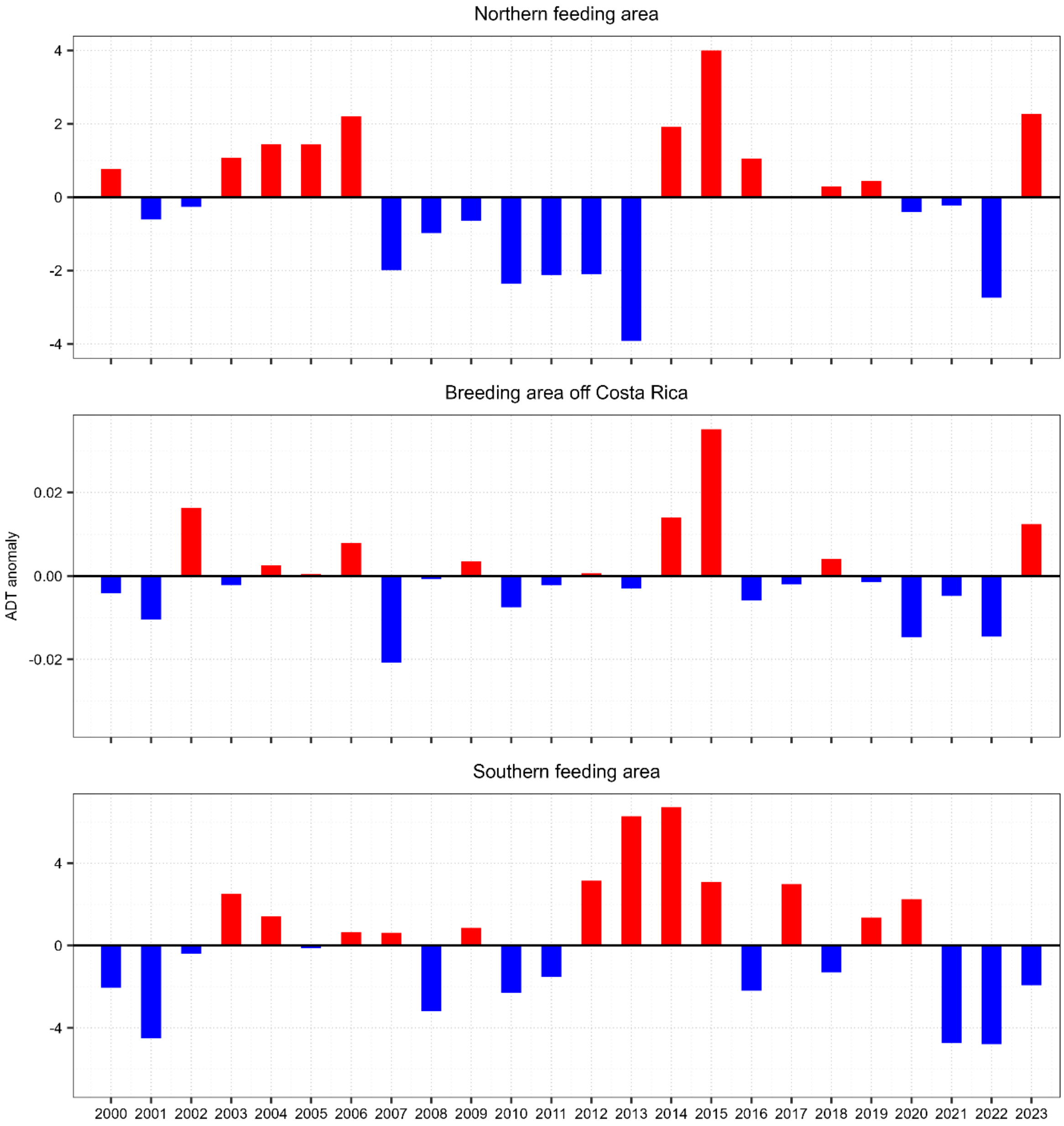

FIGURE S3. Historical anomalies of Absolute Dynamic Topography (ADT) across the northern and southern feeding areas and the breeding area off Costa Rica.

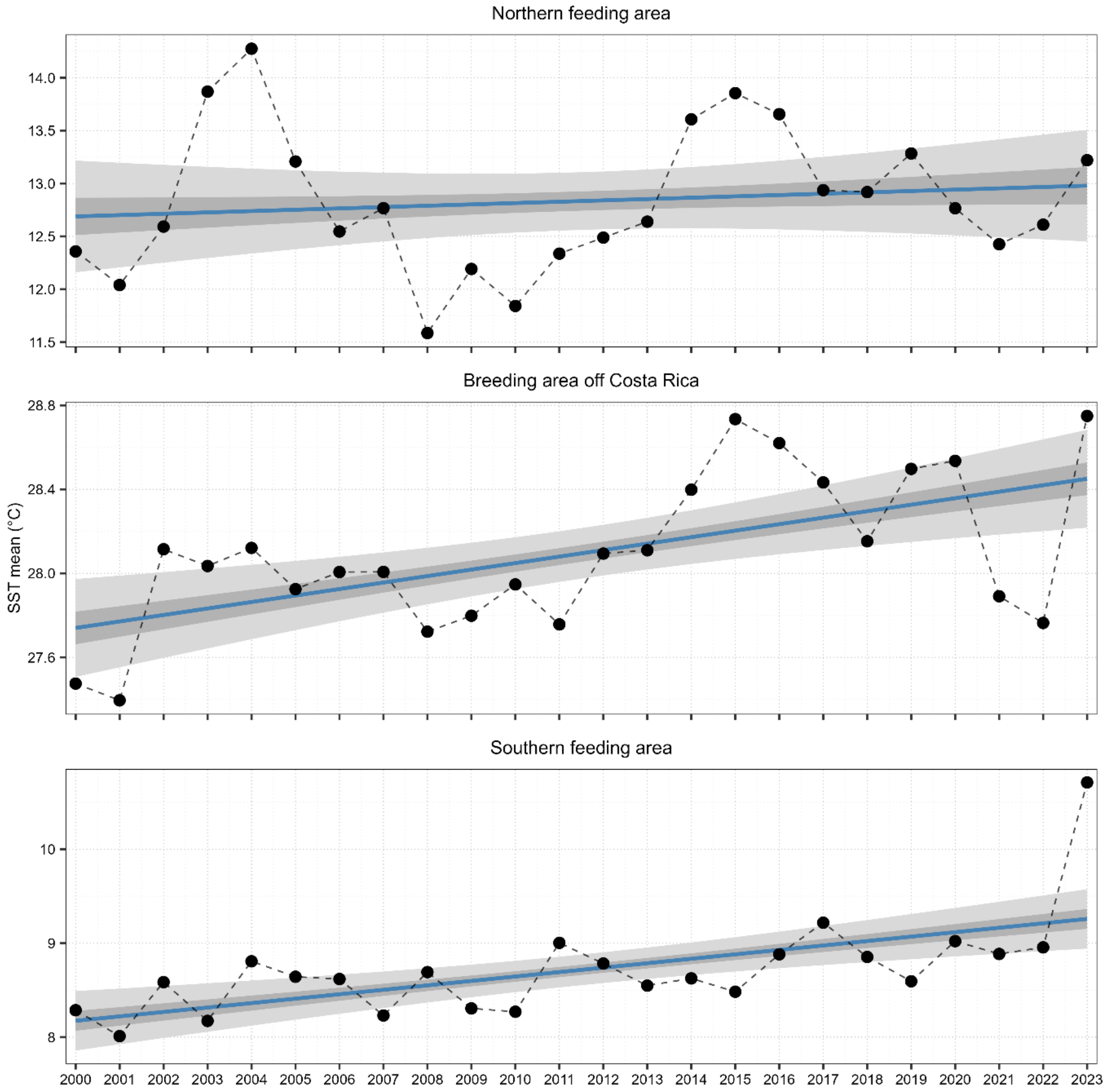

FIGURE S4. Time series of historical averages of sea surface temperature (SST) across the three regions. Mean SST values for humpback whale feeding and breeding areas are shown in black points. The northern Pacific feeding area is represented from April to August, the southern Pacific feeding area from October to February, and annual averages are shown for the breeding area off Costa Rica. Shaded gray areas represent the 75% and 95% credible intervals, and the median effect is indicated by the blue line.

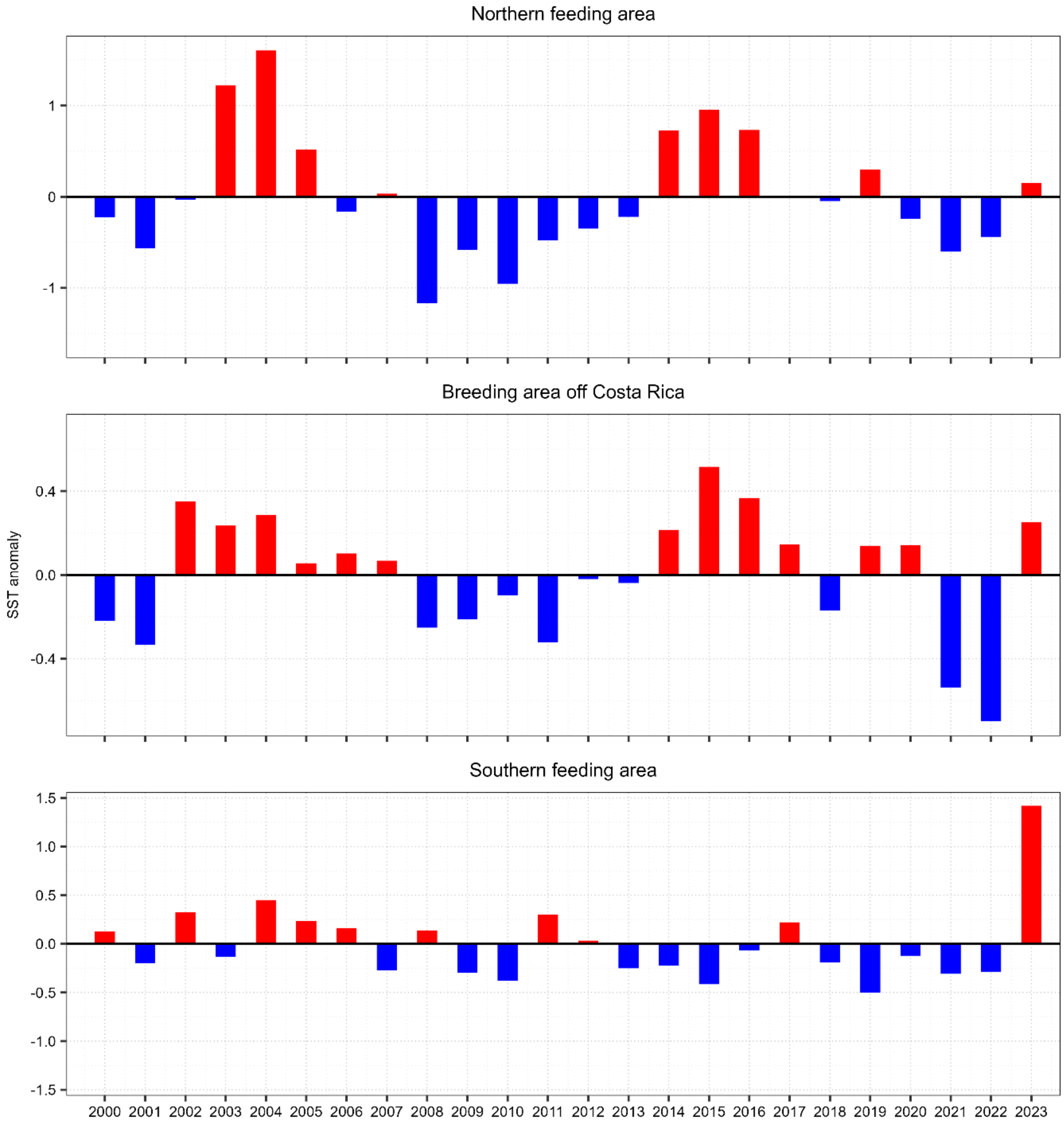

FIGURE S5. Historical anomalies of sea surface temperature (SST) across the northern and southern feeding areas and the breeding area off Costa Rica.

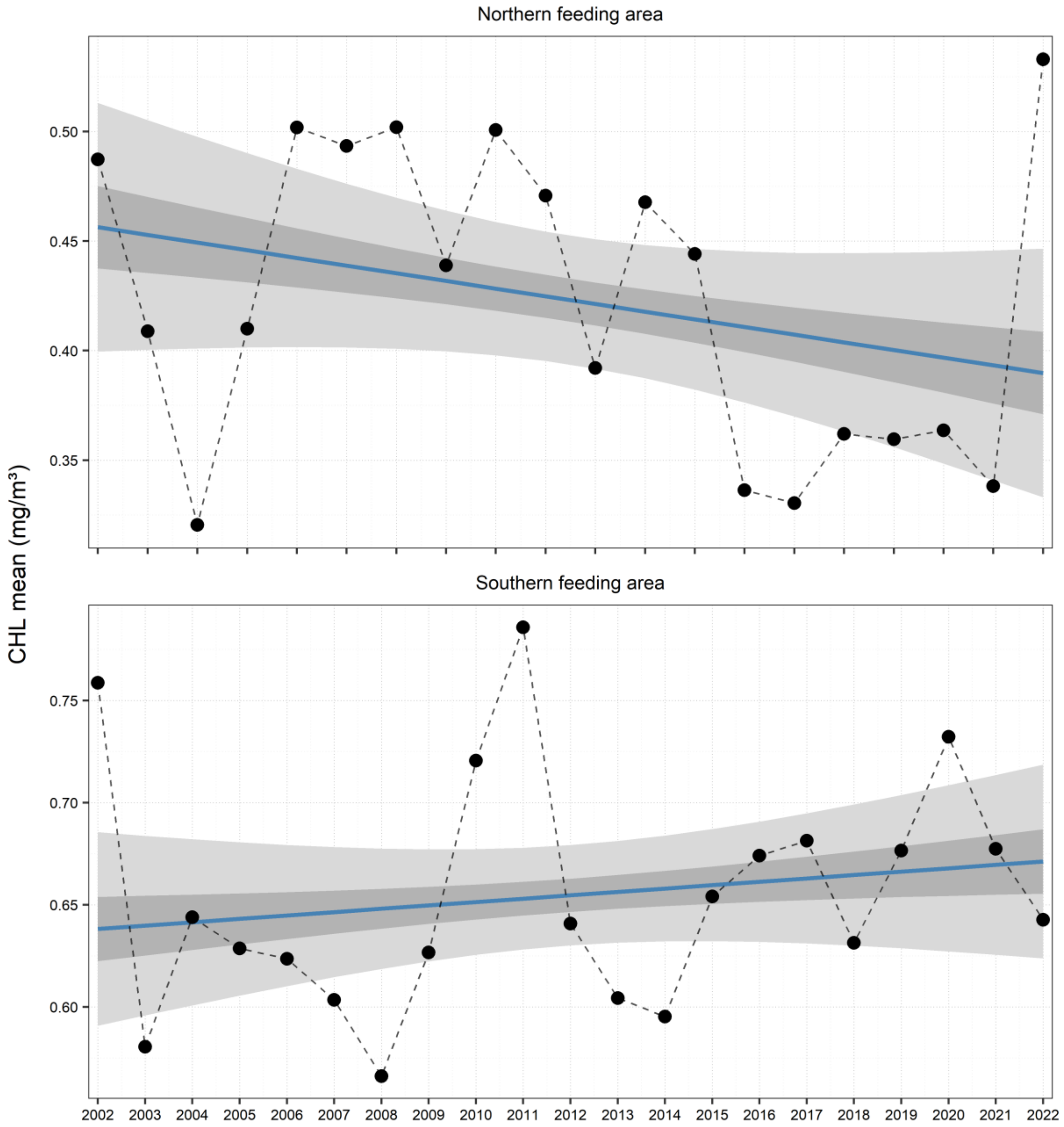

FIGURE S6. Time series of historical averages of sea surface chlorophyll-a concentration (CHL) across the feeding areas. Mean CHL values for humpback whale feeding areas are shown (black points). The northern Pacific feeding area is represented from April to August, and the southern Pacific feeding area from October to February,. Shaded gray areas represent the 75% and 95% credible intervals, and the median effect is indicated by the blue line

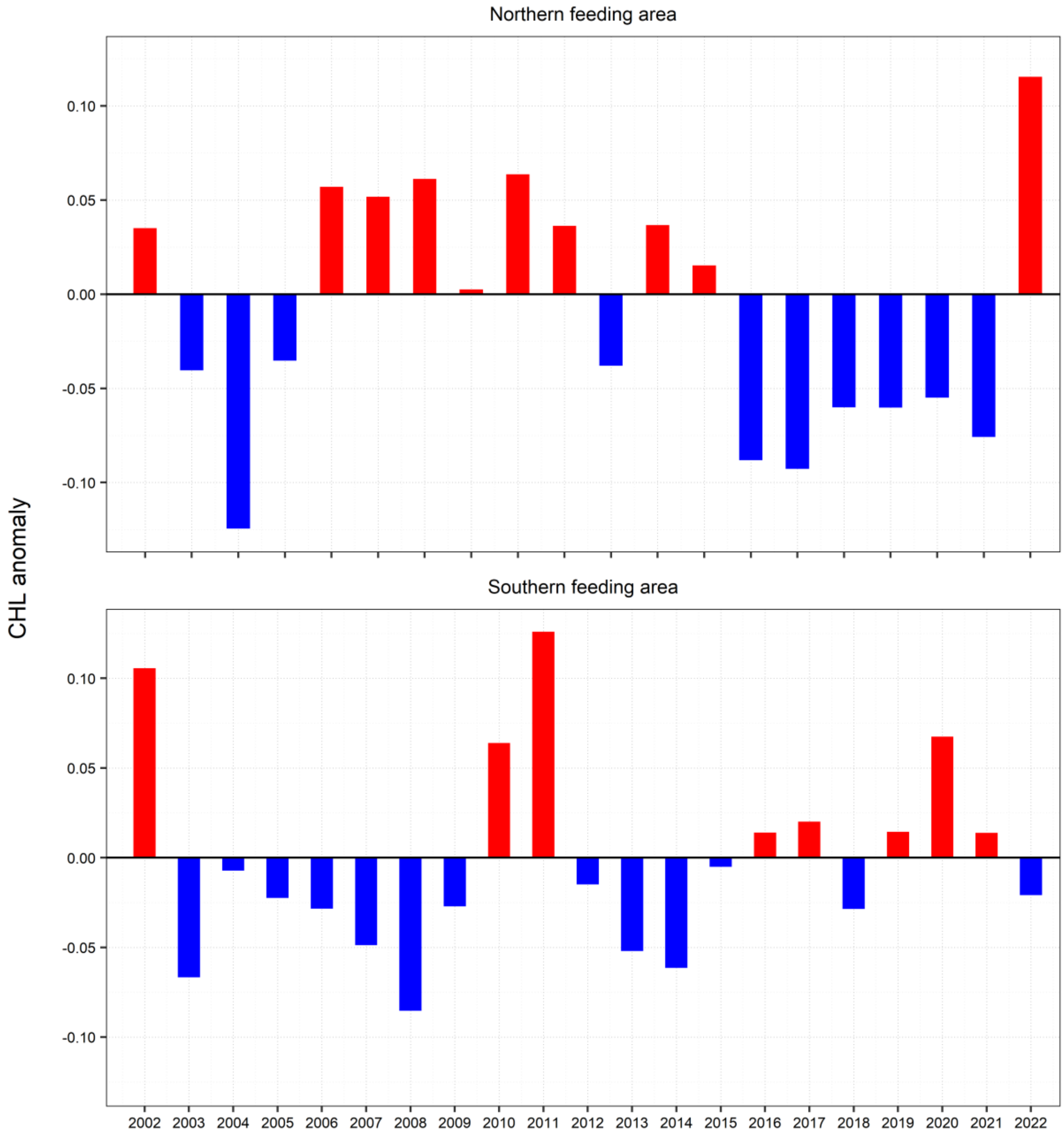

FIGURE S7. Historical anomalies of sea surface chlorophyll-a concentration (CHL) across the northern and southern feeding areas.
